# Implementing Individually Tailored Prescription of Physical Activity in Routine Clinical Care: Protocol of the Physicians Implement Exercise = Medicine (PIE=M) Development and Implementation Project

**DOI:** 10.2196/19397

**Published:** 2020-11-02

**Authors:** Leonie A Krops, Adrie J Bouma, Femke Van Nassau, Joske Nauta, Inge van den Akker-Scheek, Willem JR Bossers, Johan Brügemann, Laurien M Buffart, Ronald L Diercks, Vincent De Groot, Johan De Jong, Caroline S Kampshoff, Marike Van der Leeden, Hans Leutscher, Gerjan J Navis, Salome Scholtens, Martin Stevens, Morris A Swertz, Sacha Van Twillert, Joeri Van der Velde, Johannes Zwerver, Lucas HV Van der Woude, Willem Van Mechelen, Evert ALM Verhagen, Helco G Van Keeken, Hidde P Van der Ploeg, Rienk Dekker

**Affiliations:** 1 Department of Rehabilitation Medicine University Medical Center Groningen University of Groningen Groningen Netherlands; 2 Research Group Applied Sports Science School of Sports Studies Hanze University of Applied Sciences Groningen Groningen Netherlands; 3 Department of Public and Occupational Health, Amsterdam Public Health Institute Amsterdam University Medical Centers Vrije Universiteit Amsterdam Amsterdam Netherlands; 4 Department of Orthopedic Surgery University Medical Center Groningen University of Groningen Groningen Netherlands; 5 The Lifelines Cohort Study Roden Netherlands; 6 Department of Cardiology University Medical Center Groningen University of Groningen Groningen Netherlands; 7 Department of Physiology Radboud University Medical Center Nijmegen Netherlands; 8 Amsterdam Movement Sciences, Department of Rehabilitation Medicine Amsterdam University Medical Centers, Vrije Universiteit Amsterdam Amsterdam Public Health Research Institute Amsterdam Netherlands; 9 Department of Medical Oncology, Cancer Center Amsterdam Amsterdam University Medical Centers Vrije Universiteit Amsterdam Amsterdam Netherlands; 10 Knowledge Centre for Sport & Physical Activity Ede Netherlands; 11 Department of Internal Medicine University Medical Center Groningen University of Groningen Groningen Netherlands; 12 Genomics Coordination Center University Medical Center Groningen University of Groningen Groningen Netherlands; 13 Center of Expertise on Quality and Safety University Medical Center Groningen University of Groningen Groningen Netherlands; 14 Center for Human Movement Sciences University Medical Center Groningen University of Groningen Groningen Netherlands; 15 Sports Valley Sports Medicine Gelderse Vallei Hospital Ede Netherlands; 16 School of Human Movement and Nutrition Sciences Faculty of Health and Behavioural Sciences University of Queensland Brisbane Australia; 17 Division of Exercise Science and Sports Medicine Department of Human Biology, Faculty of Health Sciences University of Cape Town Cape Town South Africa; 18 Physiotherapy and Population Sciences School of Public Health University College Dublin Dublin Ireland

**Keywords:** clinicians, Exercise is Medicine initiative, physical activity, exercise referral, conventional treatment, hospital care

## Abstract

**Background:**

The prescription of physical activity (PA) in clinical care has been advocated worldwide. This “exercise is medicine” (E=M) concept can be used to prevent, manage, and cure various lifestyle-related chronic diseases. Due to several challenges, E=M is not yet routinely implemented in clinical care.

**Objective:**

This paper describes the rationale and design of the Physicians Implement Exercise = Medicine (PIE=M) study, which aims to facilitate the implementation of E=M in hospital care.

**Methods:**

PIE=M consists of 3 interrelated work packages. First, levels and determinants of PA in different patient and healthy populations will be investigated using existing cohort data. The current implementation status, facilitators, and barriers of E=M will also be investigated using a mixed-methods approach among clinicians of participating departments from 2 diverse university medical centers (both located in a city, but one serving an urban population and one serving a more rural population). Implementation strategies will be connected to these barriers and facilitators using a systematic implementation mapping approach. Second, a generic E=M tool will be developed that will provide tailored PA prescription and referral. Requirements for this tool will be investigated among clinicians and department managers. The tool will be developed using an iterative design process in which all stakeholders reflect on the design of the E=M tool. Third, we will pilot-implement the set of implementation strategies, including the E=M tool, to test its feasibility in routine care of clinicians in these 2 university medical centers. An extensive learning process evaluation will be performed among clinicians, department managers, lifestyle coaches, and patients using a mixed-methods design based on the RE-AIM framework.

**Results:**

This project was approved and funded by the Dutch grant provider ZonMW in April 2018. The project started in September 2018 and continues until December 2020 (depending on the course of the COVID-19 crisis). All data from the first work package have been collected and analyzed and are expected to be published in 2021. Results of the second work package are described. The manuscript is expected to be published in 2021. The third work package is currently being conducted in clinical practice in 4 departments of 2 university medical hospitals among clinicians, lifestyle coaches, hospital managers, and patients. Results are expected to be published in 2021.

**Conclusions:**

The PIE=M project addresses the potential of providing patients with PA advice to prevent and manage chronic disease, improve recovery, and enable healthy ageing by developing E=M implementation strategies, including an E=M tool, in routine clinical care. The PIE=M project will result in a blueprint of implementation strategies, including an E=M screening and referral tool, which aims to improve E=M referral by clinicians to improve patients’ health, while minimizing the burden on clinicians.

## Introduction

Over the past century, life expectancy has increased to over 80 years in many developed countries, largely because of reduced mortality rates associated with infectious diseases, childbirth, and malnutrition [1,2]. At the same time, a global pandemic of physical inactivity has occurred that contributes to dramatic increases in lifestyle-related chronic diseases [3,4]. As a result, the increase in life expectancy has not been accompanied by a comparable increase in the number of years spent in good health [2]. To increase these healthy life years, physical activity (PA) plays a crucial role in reducing the risk of a range of noncommunicable diseases. Because of its health benefits, PA improves daily life functioning, wellbeing, and quality of life and reduces healthcare costs [5]. A recent study has conservatively estimated the global financial burden of physical inactivity to be US $68 billion annually [6]. Hence, improving PA has been identified as a “best buy” for public health [5].

In the general population, the association between PA and morbidity is inverse and curvilinear; the biggest health gain can be achieved by getting inactive people to move [7]. Meeting PA guidelines of 150 minutes of moderate to vigorous intensity PA per week and muscle-strengthening activities 2 times per week is internationally recommended [5,8,9]. Also, in patients, increased PA leads to improved health and fitness, leading to maintenance of functional independence and improved quality of life [10,11]. Besides its effects on morbidity, PA is effective in preventing mortality in different patient groups. A recent meta-analysis of 305 RCTs with 339,274 participants indicated that exercise, being a specific subset of PA that is planned, structured, and repetitive, had at least similar mortality benefits to those of drug interventions in patients with lifestyle-related chronic diseases (eg, coronary heart disease, stroke, heart failure, and prediabetes) [12]. In cancer patients, meeting PA guidelines has been shown to reduce the relative risk of mortality up to almost 40%-50% [13].

The prescription of PA in clinical care has been advocated worldwide through the paradigm of “exercise is medicine” (E=M) [14-16]. Prescribing PA to patients can be used to prevent, manage, and cure various lifestyle-related chronic diseases and to prevent the development of both primary and secondary chronic diseases [17-20]. E=M differs from conventional medicine in that it treats the underlying physiological causes of disease and patients become active in managing their own health, whereby it fits the new definition of health as “the ability to adapt and self-manage” [21]. Initiatives for the implementation of E=M in primary care exist [22,23], but it has been suggested that E=M should also be part of the hospital care system (secondary and tertiary care) in terms of treatment and prescription [24,25]. E=M has great potential because of the authority and important role that clinicians play [26,27]. Periods of impaired health and time spent in the hospital can make patients receptive to behavioral change, creating teachable moments to counsel patients how to implement a physically active lifestyle [28].

Several challenges for implementing E=M at the individual clinician level are described in the literature [29]. First, some clinicians are, due to their medical training, much more likely to opt for the prescription of medication or choose other treatment options, such as surgery, rather than prescribing E=M, which is not part of regular medical training currently [30,31]. Second, clinicians are often unaware of the possibilities for, or feel uncomfortable, referring their patients to exercise professionals in or outside the hospital [29]. Third, clinicians may experience time constraints to discuss E=M with patients [30] or lack feasible tools to prescribe E=M in day-to-day care [29]. Accordingly, if clinicians prescribe E=M, it is often in the form of abstract, brief, general advice, rather than concrete, tailored prescription. To increase uptake of PA prescriptions by patients, robust implementation of personalized E=M into routine clinical care is needed.

A recent overview paper by Bowen et al [29] described several opportunities to deal with these challenges to optimally facilitate sustainable implementation of E=M in routine clinical care. They suggested that E=M can be implemented in routine clinical care via an E=M tool in electronic medical records (EMRs). Such a tool could assess patients’ current PA level, cardiometabolic risk, and overall health status related to PA, after which a clinical decision algorithm could help tailor PA prescription for each individual patient [29]. By generating a tailored PA prescription, such a tool has the potential to facilitate the implementation of E=M without requiring extensive knowledge on local PA facilities or major time investment by clinicians.

This paper describes the rationale and design of the Physicians Implement Exercise = Medicine (PIE=M) project, in which 2 diverse university medical centers in the Netherlands (both located in a city, but one serving an urban population and one serving a more rural population) will work towards implementing E=M into routine clinical care. PIE=M will address the following 3 objectives. First, we will determine levels and determinants of PA in different patient populations and compare this to the healthy population, in order to study the need for E=M. We will also determine the current implementation status of E=M and facilitators and barriers of E=M implementation for clinicians and hospital managers in selected clinical departments of both university medical centers. Based on these barriers and facilitators, a tailored set of implementation strategies will be selected to stimulate the implementation of E=M in the 2 university medical centers involved. Second, we will develop an E=M tool that will provide individually tailored PA prescriptions and referrals, in co-creation with clinicians who are the end users of this tool. Third, we will test the feasibility of the new E=M implementation strategies, including the E=M tool, when implemented in routine work processes of clinicians in 2 university medical centers.

## Methods

Given its multidisciplinary nature, the PIE=M project will be performed by a large consortium including all relevant stakeholders. This consortium consists of clinicians working in the departments of Rehabilitation Medicine and Medical Oncology of the Amsterdam University Medical Centers (both Amsterdam UMC location VUmc) and the departments of Rehabilitation Medicine, Orthopedics, and Sports Medicine of the University Medical Center Groningen (all UMCG). Moreover, researchers, professionals in technical information technology, implementation experts, sports organizations, municipalities, lifestyle professionals, and patient representatives are involved in the consortium. Throughout this paper, the term “clinicians” refers to physicians and residents working in secondary and tertiary health care. Given the participating medical departments in the PIE=M project, clinicians will be rehabilitation physicians, oncologists, orthopedists, and sports physicians. The health benefits of PA are not assumed to be diagnosis-specific, whereby the PIE=M project targets patients suffering from different physical diseases or disabilities. Throughout this paper, the term “patients” thereby refers to people with different physical diagnoses who are treated within the participating medical departments. Patients, for instance, have musculoskeletal disorders, multiple sclerosis, diabetic neuropathy, osteoarthritis, or cancer.

**Figure 1:**
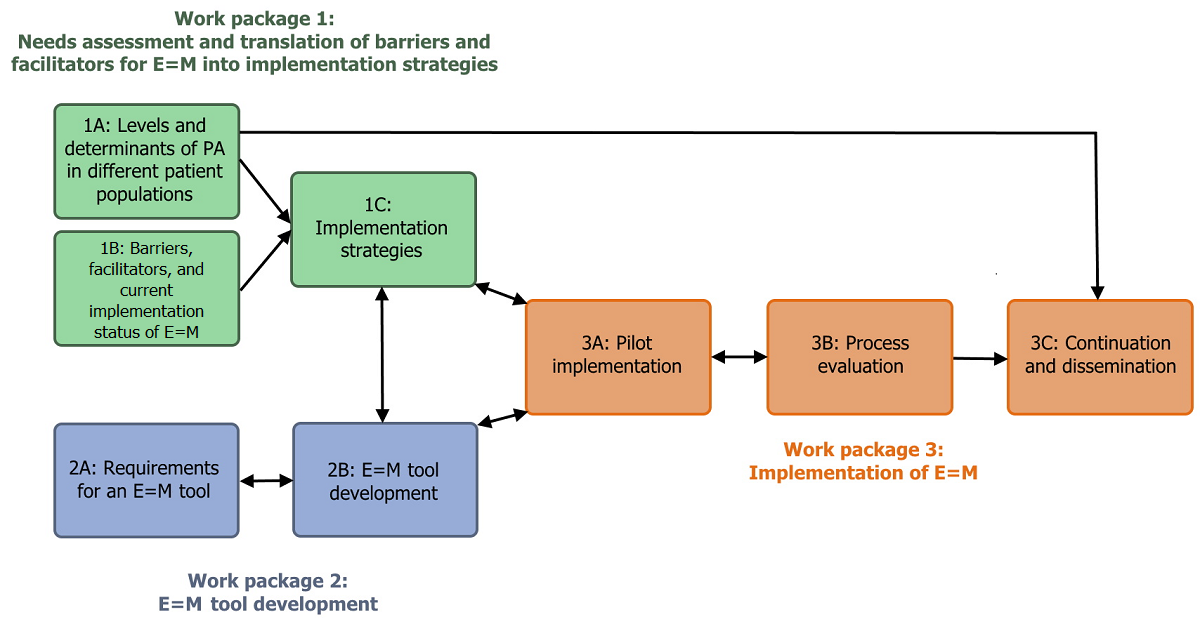
Visual representation of the 3 work packages of the Physicians Implement Exercise = Medicine (PIE=M) project. PA: physical activity; E=M: Exercise = Medicine.

The PIE=M project consists of 3 interrelated work packages, visually presented in Figure 1. The determinants that will be assessed in the different work packages of the PIE=M project are presented in Table 1, separated at the individual clinician, strategy, and patient levels.

**Table 1:** Determinants that will be assessed in the different work packages of the Physicians Implement Exercise = Medicine (PIE=M) project.

Work packages		Individual clinician level (methods used)	Strategy level (methods used)	Patient level (methods used)
**Work package 1**				
	1A	N/A^a^	N/A	Level of PA in different patient populations (Lifelines) Determinants of PA in different patient populations (Lifelines) Health benefits of PA in different patient populations (Lifelines)
	1B	Current implementation status of E=M^b^ (questionnaire and interview) Barriers and facilitators for implementation of E=M (questionnaire and interview)	Barriers and facilitators for implementation of E=M (interview)	N/A
	1C	Implementation strategies (implementation mapping)	Implementation strategies (implementation mapping)	Patients’ perspective on implementation strategies (panel discussion)
**Work package 2**				
	2A	Requirements for an E=M tool (questionnaire and interview)	Requirements for an E=M tool (questionnaire and interview)	N/A
	2B	Reflection on E=M tool designed by information technology (testing and feedback phases)	Reflection on E=M tool designed by information technology (testing and feedback phases)	Reflection on E=M tool designed by information technology (testing and feedback phases)
**Work package 3**				
	3A	Implementation of PIE=M implementation strategies (stepwise implementation)	N/A	N/A
	3B	Reach, effectiveness, adoption, implementation, and maintenance of PIE=M implementation strategies (questionnaires, logbook, field notes, interviews) Transferability of PIE=M implementation strategies to other hospitals (interviews with clinicians working in nonparticipating hospitals)	Effectiveness, implementation, and maintenance of PIE=M implementation strategies (interviews)	Experiences with the E=M implementation (interview)
	3C	N/A	Recommendations for implementation and maintenance of E=M prescription (blueprint)	N/A

^a^N/A: not applicable.

^b^E=M: exercise=medicine.

### Work Package 1: Needs Assessment and Translation of Facilitators and Barriers for E=M Into Implementation Strategies

Work package 1 will determine the need for the implementation of E=M by studying levels and determinants of PA in different patient populations and the current implementation status of E=M in the involved clinical departments. Barriers and facilitators regarding implementation of E=M will be investigated, and implementation strategies will be matched to these barriers and facilitators.

#### 1A: Levels and Determinants of PA in Different Patient Populations

To better map the need for E=M, we will identify levels of PA and the factors associated with PA in different patient populations and compare these to the healthy adult population. To do so, data from the Lifelines prospective cohort study [32] will be used. Lifelines is a multidisciplinary, prospective, population-based cohort study with a 3-generation design that studies the health and health-related behaviors of 167,729 people living in the North of The Netherlands. To recruit participants, the majority of all general practitioners in the 3 northern provinces of The Netherlands invited all their patients between the ages of 25 and 50 years, except people with very severe health conditions with a life expectancy <5 years and people with insufficient knowledge of the Dutch language. Participants invited their family members in order to develop a 3-generation cohort. Moreover, inhabitants could register themselves via the website. Inclusion stopped when the target number of 165,000 participants was reached, which was assumed to be a representative sample [32]. Lifelines data that are of special interest for the PIE=M project include demographics, lifestyle behavior (eg, PA, sitting time, smoking, nutrition, and sleep), health outcomes (eg, blood and urine biomarkers), wellbeing, and mortality. The Lifelines cohort includes healthy people as well as people with disabilities or chronic diseases, such as people with osteoarthritis, diabetes, stroke, rheumatoid arthritis, amputation, cancer, or multiple sclerosis. The Lifelines cohort performed a baseline assessment during 2007-2014. A second assessment was done during 2014-2018. The analyses will use data from the baseline and second screening of Lifelines and will be performed in SPSS 23. The 3 aims of the proposed analyses are: (1) to determine PA levels assessed with the validated Short QUestionnaire to Assess Health enhancing PA (SQUASH) [33] in different patient populations treated within the departments participating in the PIE=M project (eg, stroke, cancer, osteoarthritis) in relation to the “healthy” adult Lifelines population, (2) to determine factors associated with a physically (in)active lifestyle in different patient populations in relation to the healthy adult Lifelines population, and (3) to determine health benefits of PA in different patient populations in relation to the healthy adult Lifelines population. The results will be used to better inform clinicians on the PA levels of their patient population, the factors associated with PA, and the health benefits of PA for their patient population, which can inform their E=M practice.

#### 1B: Barriers, Facilitators, and Current Implementation Status of E=M

The current status of E=M implementation and the barriers and facilitators towards implementation of E=M in Dutch routine clinical care will be assessed using a mixed-methods approach. Clinicians (physicians, residents, physician assistants, nurse practitioners, and therapists) working in the aforementioned participating clinical departments will be invited to complete a short questionnaire. This questionnaire will include questions on the current provision of E=M-prescription, familiarity of clinicians with the Dutch national PA guidelines [9], and perception on roles and responsibilities for prescribing E=M. The questionnaire will be constructed based on questionnaires previously used among general practitioners on the same topic in the Netherlands [34].

Responding clinicians will be asked for their willingness to participate in a semistructured interview to get a more in-depth understanding of the barriers and facilitators towards implementing E=M and the added value and needed content of an E=M tool. The interview guide will expand on the model of Fleuren et al [35] with items concerning E=M as intervention and characteristics of the users, target group, organization, and sociopolitical context. Interviews will be performed until data saturation to ensure all relevant barriers and facilitators are identified. Interviews will be transcribed verbatim and analyzed using a framework analysis [36]. Strategy-level hospital managers of the same clinical departments will be interviewed to study factors that can influence the implementation of E=M prescription in clinical care from a more organizational point of view. For these interviews with managers, small adaptations will be made to the interview questions used in the clinician interviews. Data analysis methods will be similar as those for the interviews with clinicians. Clinicians and strategy-level managers of both hospitals will be interviewed to identify differences between the 2 hospitals.

#### 1C: Implementation Strategies

To address the identified facilitators and barriers, we will develop a set of tailored implementation strategies specific for each hospital. A systematic implementation mapping approach using strong stakeholder participation will be used to match implementation strategies to identified barriers and facilitators [37]. Theory and evidence-informed strategies from the taxonomy of behavior change methods [38] and the Effective Practice and Organization of Care taxonomy [39] will be used. A priori, motivating, and educative strategies and a digital tool like the proposed E=M tool are needed [29]. Selected methods can be, for instance, the distribution of educational material (flyers, posters, video), demonstration meetings, an overview of local PA facilities, introduction of local implementation leaders (eg, influential clinicians at the different departments), and prompts. Although the primary focus of the PIE=M project will be the E=M referral by clinicians, a panel discussion with patient representatives will be organized to reflect on the developed strategies for implementing E=M from a patient perspective.

### Work Package 2: E=M Tool Development

Within the second work package, a generic assistive tool will be developed for clinicians. This tool provides tailored E=M prescription and referral to local professionals in or outside the hospital. The E=M tool is based on a clinical decision algorithm that integrates individual patient characteristics (assessed by a short questionnaire) and existing health norms.

#### 2A: Requirements for an E=M Tool

End user (clinician) requirements for an E=M tool will be identified using the results derived from a questionnaire and semistructured interviews with clinicians and strategy-level hospital managers from the clinical departments involved at both university medical centers. Aspects that will be investigated are, for instance, patient characteristics and health norms for which the E=M prescription should be tailored, technical aspects of the tool, and presentation of the E=M prescription. To differentiate among individual patients, it is a priori suggested to tailor the E=M prescription based on patients’ motivation regarding PA and exercise. These aspects will be translated into the exact content of the clinical decision algorithm of the E=M tool (eg, which patient characteristics will be assessed, health norms applied) by the researchers. In addition, specific local information will be gathered for contextual use of the E=M tool, like local E=M referral options and suitability of different patient groups within the medical specialty. In a blueprint for implementing E=M, the actions needed for contextual adaptation will be thoroughly described to facilitate use of E=M in subsequent implementation projects.

#### 2B: E=M Tool Development Process

A generic tool, linked to the EMR, will be developed using an iterative interactive design process to generate a tailored E=M prescription, using individual patient and health condition characteristics. During the design process, clinicians, managers, information technology experts, implementation experts, and patient representatives will reflect on the design of the E=M tool to ensure that the E=M tool will fit the needs of stakeholders (co-creation) and that the E=M tool is consumer friendly and fulfills the general data protection regulation. Design phases (in co-creation with information technology experts) will be alternated by testing and feedback phases in co-creation with clinicians of the clinical departments involved. The E=M tool will be implemented into digital systems that link to the EMR systems (Epic Systems Corporation, Verona, WI) that are currently used by the participating departments.

### Work Package 3: Implementation of E=M

The third work package will determine the feasibility of implementing E=M, by implementation of the set of implementation strategies formulated in work package 1 together with the E=M tool designed in work package 2 (herein PIE=M implementation strategies). Pilot studies will run in 4 clinical departments of the 2 university medical centers: Rehabilitation Medicine and Orthopedics (UMCG) and Rehabilitation Medicine and Medical Oncology (Amsterdam UMC location VUmc). In general, these clinical departments have a focus on PA, which makes them suitable to pilot test the feasibility of the PIE=M implementation strategies.

#### 3A: Pilot Implementation

We will stepwise implement the set of implementation strategies of E=M (work package 1) including the E=M tool (work package 2), together referred to as PIE=M implementation strategies. In this stepwise implementation, pilots will be performed sequentially in the 4 clinical departments mentioned in the previous sections, whereby experiences from pilots conducted in the first departments will be taken into account in the implementation strategy in the departments to follow. Pilot studies will be performed between October 2019 and November 2020.

#### 3B: Process Evaluation

The pilot implementation will be monitored and evaluated with a learning process evaluation, which is especially useful when evaluating complex, real-world interventions [40]. A learning process evaluation integrates implementation and evaluation of interventions by iterative plan-do-study-act cycles, which makes it especially suitable for evaluating stepwise implementation processes [41]. Both contextual and explanatory factors related to implementation will be determined, as well as their effect on implementation outcomes across organizations. This makes a learning process evaluation a suitable method for our multicenter pilot implementation [40]. The process evaluation has 3 specific objectives. First, Reach, Effectiveness, Adoption, Implementation, and Maintenance (RE-AIM) of the implementation of E=M in routine clinical care are investigated [42,43]. Operationalization of the RE-AIM model is provided in Table 2. Reach of implementing E=M will be determined by the absolute number, proportion, and characteristics of patients who are actually participating in the E=M pilot, relative to those eligible for participation. Effectiveness is defined as the impact of the set of PIE=M implementation strategies (work packages 1 and 2) on the perceived successfulness of E=M referral and as satisfaction with implementing E=M using the implemented set of PIE=M strategies (work packages 1 and 2). Adoption is operationalized as the absolute number, proportion, and representativeness of departments and clinicians who are willing to participate in the pilot, relative to those invited for participation. Implementation is operationalized as fidelity, adaptations to the PIE=M implementation strategies, and experiences with the implementation. Maintenance is operationalized as the extent to which the implementation of E=M has become part of routine care in the participating departments. The second aim of the process evaluation is to identify success and failure factors for clinicians regarding the process of implementing E=M using the proposed PIE=M implementation strategies (work packages 1 and 2). Third, recommendations for improvement of E=M in routine clinical care are investigated.

**Table 2:** Operationalization and methods used in the process evaluation following the RE-AIM framework (work package 3B)

Operationalization		Questionnaire clinician (baseline)	Questionnaire clinician (1 month)	Questionnaire clinician (end pilot)	Interview clinician (end pilot)	Interview department manager (end pilot)	Interview lifestyle coach (end pilot)	Logbook usage E=M-tool	Patient interview	Interview with non-participating hospitals	Field notes
**Reach^a^**											
	Sources and procedures for recruitment of participants, and reported reasons for (non-) participation in E=M		X	X	X	X			X		X
	Characteristics of profiled patient population per department (e.g. age, health condition)							X			
**Effectiveness^b^**											
	The impact of the PIE=M innovation strategies on perceived successful referral by clinicians		X	X	X	X	X				
	Satisfaction with the PIE=M innovation strategies among patients		X	X	X	X	X		X		
	Satisfaction with the PIE=M innovation and implementation strategies among clinicians		X	X	X	X					
	Satisfaction with the PIE=M innovation and implementation strategies among managers					X					
**Adoption^c^**											
	Characteristics of participating departments (e.g. size, patients)					X					X
	Characteristics of participating clinicians	X									
**Implementation^d^**											
	Fidelity and adaptations to the core principles of the PIE=M implementation strategies		X	X				X			
	Views and experiences with implementation of the PIE=M implementation strategies by clinicians		X	X	X	X					
	Recommendations for improvement of E=M in routine clinical care and suggestions for future implementation		X	X	X	X					
	Success and failure factors to the implementation of the PIE=M implementation strategies		X	X	X	X					
**Maintenance^e^**											
	Maintenance of the implementation of the PIE=M implementation strategies			X	X	X	X				
	Extent to which other non-involved (university) hospitals are willing to implement the PIE=M implementation strategies									X	

^a^The absolute number, and proportion of patients who are willing to participate in E=M.

^b^The impact of the PIE=M innovation strategies on perceived successful referral.

^c^The absolute number, proportion, and representativeness of settings and clinicians who are willing to initiate a program.

^d^The clinicians’ implementation of the key components of the PIE=M implementation strategies.

^e^The extent to which the PIE=M implementation strategies become part of the routine in the participating departments.

Data will be collected using a mixed-methods approach (Table 2). All participating clinicians will be invited for a baseline questionnaire as well as 2 follow-up questionnaires (1 month after baseline and at the end of the pilot). The questionnaire, which was constructed for this study, will include questions on demographic information (eg, age, years of experience, own lifestyle behavior), determinants for implementation, and perceived impact, satisfaction, and experiences with implementing E=M using the set of PIE=M implementation strategies (work packages 1 and 2). In the last questionnaire, a question will be added about the extent to which E=M has become routine care and whether clinicians have suggestions for further improvement of the set of PIE=M implementation strategies. Moreover, the use of the newly developed E=M tool (work package 2) will be tracked using a logbook completed by the participating clinicians during their consultations and field notes made by the researchers.

During and at the end of the pilot, semistructured interviews will be organized with a subsample of the involved clinicians, involved department managers, and eventually other relevant stakeholders, such as lifestyle coaches, in case they have been involved in the pilot departments. During these interviews, we will reflect further on the perceived impact of and satisfaction with the set of PIE=M implementation strategies and the extent to which E=M has become routine care. A subsample of patients who were selected for participating in the pilot will be invited to participate in a short structured face-to-face interview to evaluate their experiences with the E=M implementation. Lastly, clinicians working in nonparticipating hospitals will be invited to participate in an interview to explore the transferability of the set of implementation strategies including the E=M tool to other departments and hospitals in the Netherlands. All semistructured interviews will be transcribed verbatim and analyzed using a framework analysis approach based on the model by Fleuren et al [35].

#### 3C: Continuation and Dissemination

During the implementation process, plans for securing the implementation of E=M prescription in the clinical departments involved will be determined, in collaboration with representatives from the clinical departments. The process evaluation will result in recommendations for the implementation and maintenance of E=M prescription using the set of proposed PIE=M implementation strategies in other clinical departments or other hospitals. These recommendations will be recorded in a blueprint that can guide the implementation of E=M in other clinical departments or other hospitals. Some of the implementation strategies will be generic and applicable in different clinical departments in different hospitals. However, since implementation is highly context-dependent, part of the implementation strategies will be tailored to the specific context of the department and hospital. In addition to generic recommendations, the blueprint will hereby consist of a stepwise procedure to select applicable context-specific implementation strategies.

### Patient and Public Involvement

The primary target population of the PIE=M study is clinicians. Clinicians are involved in the project consortium and thereby involved throughout all steps of the project. Also, patients and other public stakeholders (eg, local sports organizations, municipalities) are involved throughout all steps of the PIE=M project, starting already during the writing of the research proposals. Both patients and public organizations are involved in the design, recruitment, and dissemination. Patients are asked to reflect on the burden of the E=M implementation in a panel discussion.

### Ethics and Dissemination

The study will be performed in accordance with the Declaration of Helsinki. The medical ethical committees of the UMCG and Amsterdam UMC approved the study design (METc UMCG 2017/517 and Amsterdam UMC 2018/219). Results of the PIE=M project will be published in peer-reviewed international journals and presented at conferences. Results will be used for further development of the set of implementation strategies and E=M tool, to further facilitate the implementation of E=M in participating departments and other clinical departments and hospitals.

## Results

This project was approved and funded by the Dutch grant provider ZonMW in April 2018. The project started in September 2018 and continues until December 2020 (depending on the course of the COVID-19 crisis). All data from work package 1 have been collected and analyzed and are expected to be published in the 2021. Results of work package 2 have been described. The manuscript is expected to be published in 2021. Work package 3 is currently being conducted in clinical practice in the 4 departments of the 2 university medical hospitals among clinicians, lifestyle coaches, hospital managers, and patients. Results are expected to be published in 2021.

## Discussion

This paper describes the rationale and design of the PIE=M project, which aims at sustainable implementation of E=M in routine clinical care.

PIE=M will result in a set of strategies to prescribe tailored PA advice and individual referral to local PA professionals, including an E=M tool for clinicians within the currently used EMRs [14,29]. The results of PIE=M are partly generic and partly specific to the context of the participating departments. Work package 1 will result in generic knowledge on current PA levels, and determinants of PA behavior in different patient groups will be generated. This knowledge will help to indicate the need for the implementation of E=M and could potentially be used to better tailor individual PA prescription. The implementation strategies, which are translated from the barriers and facilitators for E=M among clinicians in work package 1, are partly generic and partly specific for the context of the participating departments. The E=M-tool developed in work package 2 will consist of a generic algorithm that can be adapted to the specific local context. During this pilot, the tool will be built into the information technology systems (eg, EMRs) that are currently used in the participating departments. For future implementation in other hospitals or departments, the tool should be linked to information technology systems that are used within the department, providing opportunities for context-specific applications. The development of our E=M tool may thereby serve as an example for other decision aids in different settings aimed at E=M implementation. Work package 3 will result in a generic blueprint describing the generic implementation strategies and a stepwise procedure with tools to select applicable context-specific implementation strategies.

A strength of the PIE=M project is the multidisciplinary collaboration between patients, clinicians working in various clinical departments from 2 university medical centers, researchers from different disciplines, professionals in technical information technology and universities of applied sciences, municipalities, and lifestyle professionals. Another strength of this study is that 2 different hospitals are involved in this project, of which one is located in a very urban area and one is located in a city but serves a more rural area. This involvement enables comparison between the implementation in different organizations and different geographical contexts. We expect this comparison will enhance the recommendations for implementation in other hospitals, which will be described in the blueprint. It should be considered that both hospitals involved are university medical centers providing specialized top clinical care, which might limit the generalizability of the findings to smaller community hospitals. It should also be considered that the participating clinical departments may have a stronger focus on the importance of PA than other clinical departments. Barriers and facilitators might differ in clinical departments with less focus on the importance of PA, which could result in different implementation strategies. However, the developed E=M tool and blueprint for implementing E=M will be generic and applicable in departments with less focus on PA. However, arguably the most important strength of the PIE=M project is the involvement of end users, both clinicians and patients. This ensures applicability in routine clinical practice. By referring patients to existing local PA facilities, both within the hospital and the community, long-term sustainability in daily practice is maximized, contributing to external validity of the study findings.

By developing and pilot testing implementation strategies for E=M, including an E=M tool, the PIE=M project represents a next step within research on implementing E=M. However, when feasibility of the implementation of E=M has been shown, large-scale implementation studies are needed in different clinical departments in different hospitals, as well as studies on the (cost) effectiveness of E=M at the patient level.

The PIE=M project addresses the potential of providing patients with PA advice to prevent and manage chronic disease, improve recovery, and promote healthy aging by developing E=M implementation strategies including an E=M tool in routine clinical care. The PIE=M project will result in a blueprint of implementation strategies, including an E=M screening and referral tool that aims to improve E=M referrals by clinicians to improve patients’ health, while minimizing the burden on clinicians.

## Appendix

Multimedia Appendix 1 Report letter of the funder (English translation).

## Abbreviations

E=M: exercise=medicine
EMR: electronic medical record
PA: physical activity
PIE=M: Physicians Implement Exercise = Medicine
RE-AIM: Reach, Effectiveness, Adoption, Implementation, and Maintenance
UMC: University Medical Center
UMCG: University Medical Center Groningen
