# Supplementary material for: Implementing Individually Tailored Prescription of Physical Activity in Routine Clinical Care: Protocol of the Physicians Implement Exercise = Medicine (PIE=M) Development and Implementation Project
Source: JMIR Res Protoc. 2020 Nov 2;9(11):e19397. doi: 10.2196/19397 (PMC7669441; doi:10.2196/19397)
Supplement: Multimedia Appendix 1 [file resprot_v9i11e19397_app1.pdf]

On October 4, 2017 you submitted a grant application to ZonMw entitled: *PIE=M; Physicians Implement Exercise is Medicine. The Implementation of individualized active lifestyle prescription in routine clinical care; E=M.* We are pleased to inform you that the committee Sports and Physical activity 2017 has given a positive assessment of your application. This means that ZonMw will award the grant to you. In this letter we explain how ZonMw arrived at this decision and what you must do before your project can commence.

Assessment
Your application was assessed in terms of quality and in terms of relevance to the programme and call. In this round of the programme ZonMw received 19 grant applications, of which 4 have been approved. The assessment procedure was carried out as follows:

Quality
To begin with the quality control panel issued a summary quality assessment of your grant application. This assessment was based on your application, an assessment of the application by reviewers and your response. The quality assessment was: **good.** The panel provided the following reasons:

The programme committee then adopted this quality assessment for the following reasons:

- The committee deems that clear project goals are formulated and a realistic time plan is made.
- The project plan is defined well.
- Personalization in data science is judged as good and challenging.

The final assessment of the quality of your grant application was: **good.**

Relevance
The programme committee also assessed the relevance of your grant application to the programme and call. The final assessment of the relevance of your grant application to the programme is: **very relevant.**
The committee based its assessment on the following arguments:

- According to the committee the project has a very high societal relevance.
- The committee deems that the consortium is very experienced and complementary to each other. The multidisciplinary collaboration is deemed very relevant by the committee. The committee indicates that the role of the physician can be described into more detail.

The committee advises to more extensive emphasize the individual prescription at the start of the project. You could consider the application of the FITT-principles and safety aspects.

On the basis of both final assessments the programme committee then prioritized all applications that could be considered for approval. On this basis ZonMw approved your application.

Funding

Amount of the grant
ZonMw's funding for your project will be for a total amount of € 397.900,-- for a maximum period of 24 months. This amount includes any payable VAT.

Grant conditions
As you know, the funding is subject to certain conditions. These grant conditions can be downloaded from the ZonMw website: www.zonmw.nl/subsidievoorwaarden
I emphasize that ZonMw can only pay an advance when all requirements for conducting research are met. Please start any procedures in time, such as a positive determination from a recognised medical research ethics committee (Medisch Ethische ToetsingsCommissie), the Dutch Central Committee on Research Involving Human Subjects (Centrale Commissie Mensgebonden Onderzoek), a projectlicense of the Central Committee Animal Experiments (Centrale Commissie Dierproeven), or a license granted in accordance with the Population Screening Act (Wet Bevolkingsonderzoek). If you are unsure whether your project requires such, please check with the relevant authorities.

Integrity

Article 2, paragraph 3 of the General Terms and Conditions Governing Grants of ZonMw implies that the nationally and internationally accepted standards regarding scientific conduct - as laid down in the Dutch Code of Conduct for Scientific Practice (VSNU, last revised October 31, 2014), or similar codes for non-university institutions - are respected. ZonMw should be informed immediately in case of (potential) violation of the aforementioned standards in a project funded by ZonMw. All relevant documents must be submitted to ZonMw upon request.

ZonMw determines that the annex “Agreement funding scientific research 2008” and the addendum, according to Article 7 of the agreement, is not fully applicable to the funding. They will be applied as similar as possible, whilst not conflicting with the ZonMw General Terms and conditions. The General Terms and Conditions are always leading. For example, ZonMw always settles financial accounts based on actual costs.

What should you do now?

Important: written confirmation within four weeks
ZonMw can provide you with an advance payment for the first project year. This is only possible, however, if you have agreed to the grant conditions and once your project has actually commenced. We would thus request you to submit the following information in writing to ZonMw before **april 26, 2018.**
You can use the enclosed information form for this purpose:

- Your agreement to the conditions applicable for allocation of the funding;
- The starting date of your project;
- The bank and reference data for the grant payments;
- With respect to approval by METC or CCD:
  - If no declaration(s) is (are) required, please send written confirmation of this.
  - If the declaration(s) is (are) required for the commencement of the project, send the declaration to ZonMw before the start of the project.
  - If the declaration(s) is (are) not required until later in the project, indicate when the declaration(s) is (are) necessary. This may be no later than one year after commencement of the project. ZonMw will then make an advance payment for the first year. ZonMw can make further advance payments only if a copy of the declaration(s) has been received.

I would like to point out that the project must commence **six months at the latest** after the date of this letter. If the project commences after this date, then our approval of your application will expire. Exceptions to this principle are possible only under very exceptional circumstances.

Public Summary
ZonMw publishes all approved projects on its website with an accessible summary. This summary is intended for a broad, interested readership whose language skills are roughly equivalent to that of pupils who have completed pre-university secondary education (VWO). For further details, please refer to the style guide at http://www.zonmw.nl/nl/over-zonmw/logo-huisstijl.
We kindly request that you submit this summary as soon as possible, but at least within four weeks after the date of this letter. For this purpose, you can use the text field in ProjectNet with the heading Public Summary, which limits you to 1,000 characters including spaces.

Progress Report
ZonMw would like to remain informed about the progress of your project. On around July 2019 the programme secretary will send you a request to submit a progress report. ZonMw applies a summary progress report that you submit half-way through the project (unless otherwise specified). In addition, you are obliged to report any interim changes to ZonMw. The changes are permitted only with the approval of ZonMw.

Utilisation of knowledge
Results from the research project can be implemented, play a part in policy making, become the next step in a scientific career or constitute the basis of a new project.
In order to gain insight in the use of the results from the study, we request information on the implementation and spread through several questions in the progress report(s) and final report. For a period of four years following the conclusion of the study you are obliged to submit all publications and results of the project into ProjectNet.
During this period you are obliged to inform ZonMw of the use of these results.

Datamanagement
To allow future reuse of data from your project, ZonMw requires every grant recipient to draw up a data management plan. You can find information about the procedures and tools at www.zonmw.nl/en/research-and-results/fair-data-and-data-management/data-management-in-your-project/ Starting from this link, follow the steps. ZonMw does not review the plans. ZonMw monitors the outcome of your data management during and at the end of your project with a number of key items for FAIR data. Please use the list for providing the information on key items at www.zonmw.nl/en/research-and-results/fair-data-and-data-management/data-management-in-your-project

Please send the data management plan and the key items (as far as you know them) as PDF files within 3 months to <e-mail programmateam>. During your project you can make changes and updates. In case you will not collect data, please inform ZonMw.

If this letter gives rise to any questions, please contact the individual listed in the letterhead. If you are dissatisfied with the way that ZonMw has handled your application, then you may submit a complaint (see below). Please mention your project number in all correspondence with ZonMw. Now that your application has been approved, the original number expires and you are now assigned the following project number: **546001002.**
